# A comparative study on vildagliptin brand and its generic equivalents using dissolution test as quality control measure tool

**DOI:** 10.1038/s41598-024-52674-4

**Published:** 2024-02-01

**Authors:** Ghadah H. Altoum, Fouza K. AL-Enazi, Mubarl M. Abudahash, Reem A. AL-Fadhli, Naif Alenzi

**Affiliations:** Research and Laboratories Sector, National Drug and Cosmetic Control Laboratories (NDCCL), Saudi Food and Drug Authority, Riyadh, Saudi Arabia

**Keywords:** Drug discovery, Drug delivery

## Abstract

Market drugs including brand or generic with poor quality, don’t meet the acceptable standard guidelines. Vildagliptin is an important antidiabetic drugs used in monotherapy or in combinations protocols for treatment of diabetes mellites. The main goal of the current study is to assess the pharmaceutical equivalence of two marketed generics of vildagliptin 50 mg tablets compared to the branded product (Galvus 50 mg). The in vitro dissolution test was used as a quality control tool to obtain the dissolution profile of vildagliptin compared to the reference drug. The results revealed that all tested samples showed dissolution behavior like standard drug. Whole samples dissolution reached after 15 min in accordance with the standard. According to the similarity factors records, tested vildagliptin samples showed a comparable dissolution to the reference drug. The current work presents an in vitro protocol for quality evaluation of recently released generic drugs.

## Introduction

**Figure 1:**
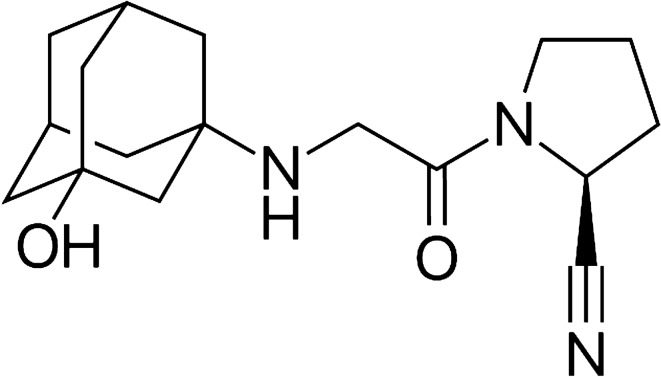
Structure of vildagliptin.

In pharmaceutical industry, Brand-name medicines (Innovator drugs) are those generated by a company and patented to increase the economic gain of being the exclusive manufacturer of such a drug^1^. When the patency of the Innovator drug product expires, another pharmaceutical manufacturing companies can apply to regulatory authorities for approval to market generic versions of the original medicines^2^. Pharmaceutical companies that produce generic drugs have not devoted much to the research and development of a new drug; they simply copied the patented formula of the original branded drug^2^. Generic products are chemically identical to the original drug and have the same active ingredients^1^. However, the inactive ingredients can be different but at a certain potency^2^. Today, the use of generic drugs has increased and many countries have introduced regulations to provide safe, effective and good quality medicines to their people^3,4^. The lower cost of generics compared to Innovator drugs has increased their existence in the local market. Therefore, it is essential to test the interchangeability of the marketed generic products using a suitable approach. The interchangeability of generics is understood to mean the possibility for their mutual replacement or replacement of the original drug in clinical practice^5^. Bioequivalence studies is considered to be costly , time consuming and involve subjecting healthy volunteers to risks of side effects^6^. Accordingly, the biopharmaceutics classification system (BCS)^7^ was developed as a substitute for the in vivo bioequivalence studies and is applicable to highly soluble drugs with known absorption rate and extent in humans, drugs with a wide therapeutic index, and orally administered drugs that are immediate release^8^. Dissolution test is a valuable quality control tool to monitor batch-to-batch consistency during drug development^9^. Additionally, dissolution testing can be used for optimization of formulations and monitoring drug stability over time^10,11^. Vildagliptin (Fig. 1) is an antidiabetic drug belonging to Dipeptidyl Peptidase-4 (DPP-4) inhibitors and approved to be used in monotherapy and combination therapy to control type 2 diabetes mellitus^12^. Vildagliptin is a BCS-I compound with high solubility and permeability^13^ and does not to have a narrow therapeutic index which account for its applicability to be assessed using in vitro dissolution approach^14^. In this work, the in vitro dissolution test is used as a quality control tool to obtain the dissolution profile and assess the equivalency of two marketed generics of vildagliptin 50 mg tablets compared to the branded product (Galvus 50 mg).

## Results and discussions

**Figure 2:**
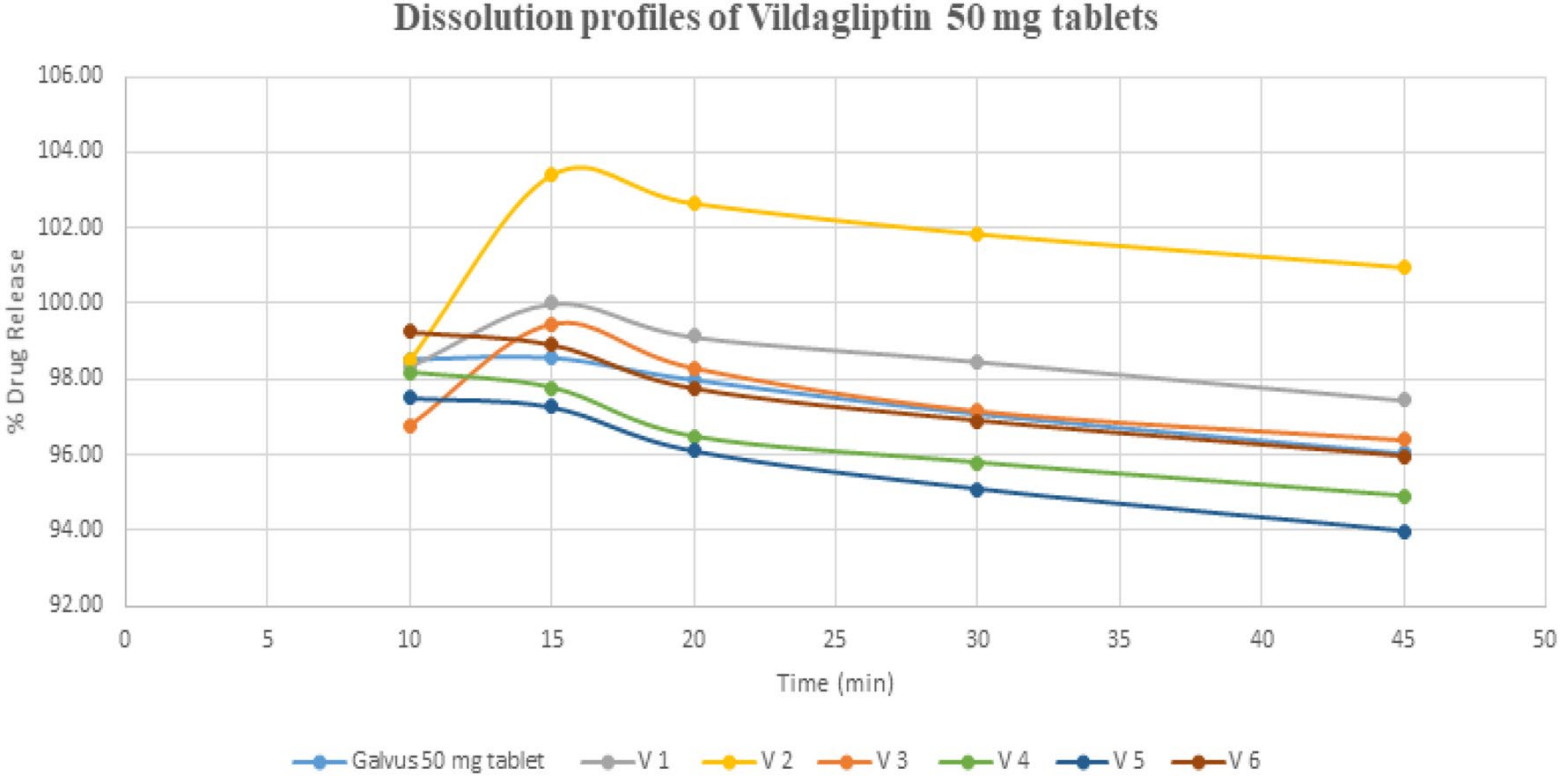
Dissolution profile of Vildagliptin 50 tables.

The dissolution pattern of six generic products was evaluated in the current experiment then compared to the dissolution profile of reference product (Galvus). As shown in Table 1, almost a similar percentage drug release is derived for all the products which confirm a fast drug release pattern accounting for the physiochemical property of vildagliptin as BCS class 1. The results of dissolution studies are presented graphically in Fig. 2. Results revealed that V-1 and V-2 samples showed the fastest drug release % reaching 100% within 15 min. Additionally for all samples, the complete dissolution reached after 20 min. As observed the first product showed a slight better and faster release than the brand and this observation was in accordance with the previous reports which is due to tablet hardness^15^.

**Table 1:** % of tested vildagliptin released at different sampling times, results were represented as mean and SD (n = 3).

Time (min)	% Drug release						
	Ref	V-1	V-2	V-3	V-4	V-5	V-6
10	98.53 ± 0.9	98.34 ± 3.5	98.48 ± 2.2	96.78 ± 2.5	98.17 ± 0.69	97.50 ± 1.48	99.26 ± 1.13
15	98.57 ± 0.94	100.01 ± 0.74	103.41 ± 1.33	99.44 ± 1.09	97.77 ± 0.66	97.27 ± 1.41	98.93 ± 0.77
20	98.00 ± 0.94	99.13 ± 0.79	102.67 ± 1.53	98.29 ± 1.02	96.48 ± 0.64	96.10 ± 1.42	97.76 ± 0.86
30	97.10 ± 0.91	98.48 ± 0.80	101.86 ± 1.52	97.16 ± 1.05	95.80 ± 0.76	95.09 ± 1.49	96.91 ± 0.78
45	96.05 ± 0.97	97.45 ± 0.89	100.97 ± 1.55	96.40 ± 1.03	94.92 ± 0.64	93.97 ± 1.46	95.96 ± 0.87

After performing the dissolution test, a mathematical comparison is calculated using the difference factor (f1) and similarity factor (f2). The FDA guidelines stated that f1 values up to 15 (0–15) and f2 values greater than 50 (50–100) ensure sameness or equivalence of generic product to the reference product^16^. All tested samples showed f1 value range from 0.33 in V-3 to 3.94 in V-2. Regarding f2 all samples ranged from 85 to 98. According to the similarity factors records, tested vildagliptin samples showed a comparable dissolution to the reference drug. As shown in Table 2, the obtained values have revealed the sameness of tested generics when compared to the reference product. Compared to the previous studies on vildagliptin dissolution test, the study done by Barden et al., showed that the release profile was satisfactory, and the dissolution was quite fast, as about 80% of vildagliptin was dissolved within 10 min and whole drug dissolution was reached within 30 min^17^. A generic drug must demonstrate adequate pharmaceutical equivalence to a reference drug that has previously received market authorization depending on clinical trials^18,19^. To decrease post marketing quality issues, a surveillance system establishment for generic products in the market is encouraged^20^. In vitro dissolution test for immediate-release solid dosage forms including tablets is employed to assure product quality and performance following changes in manufacturer parameters^21^.

**Table 2:** Calculated f1 and f2 values for tested products.

The product	f1 value	f2 value
V1	1.13	90.28
V2	3.94	67.80
V3	0.68	93.57
V4	1.05	91.39
V5	1.70	85.09
V6	0.33	98.47

## Materials and methods

### Materials and instruments

The reference product Galvus and six generic products of vildagliptin 50 mg tablet where tested (3 batches for each generic were tested for statistical analysis) were collected by the Saudi food and drug authority. The reference standard of vildagliptin was obtained from (Batch #20200808Q). Hydrochloric acid 37% was purchased from sigma Aldrich. Dissolution test was performed on Erweka dissolution tester (DT 1410). High performance liquid chromatography (HPLC) by Shimadzu was used for chemical analysis of samples.

### HPLC Instrumentation

Liquid chromatography (LC) method was carried as previously reported^17^. In brief, a liquid chromatography (LC) Shimadzu 20-A system equipped with a CBM20A system controller, LC-20AT pump, SIL20A/C auto sampler, CTO-20A/C column oven and SPD-M2OA PDA detector. The experiments were performed on an analytical column Zorbax Eclipse Plus RP-C8 (150 mm × 4.6 mm, 5 µm). The LC system was operated isocratically at 25 °C in the column oven, using a mobile-phase composed by a solution of 50 mM potassium phosphate buffer and acetonitrile (85:15 v/v), at a flowrate of 1.0 ml min^−1^, using detection at 207 nm. The pH of mobile phase was adjusted at 7.0 using phosphoric acid in the aqueous phase (by adding 0.1 mL and monitoring of pH) and then, it was done the mixture of both aqueous and organic phases. The injection volume was 20 μL. The peak areas were integrated automatically by computer using LC-Solution manager system software.

### Standard preparation

Vildagliptin standard stock solution was prepared by weighing 12.84 mg of Vildagliptin standard in 25 ml volumetric flask, then complete to volume with water. Final standard solution is obtained by pipetting 2 ml of Vildagliptin standard stock solution into a 20 mL volumetric flask then complete to volume with dissolution medium.

### In vitro dissolution test

The dissolution test of each product was performed according to the dissolution method of reference product (Innovator) Galvus. The dissolution parameters are detailed in Table 3. Where the drug release is determined using (USP) dissolution testing apparatus type 2 (paddle method) at 50 rpm. The dissolution medium volume is 0.01 N HCl, 1000 mL and maintained at 37 ± 0.5 C. Dissolution profile was obtained at specific time intervals (10, 15, 20, 30, 45) where an aliquot of 10 ml was withdrawn from the dissolution apparatus. The sample solutions were filtered using 10 μm filters. The resultant samples were chromatographically analyzed by HPLC using photodiode array detector at 210 nm. Dissolution percentage was calculated, and dissolution profile was plotted after comparing the response of vildagliptin standard solution.

**Table 3:** Dissolution Parameters included in testing each product according to USP.

Medium	0.01 N HCL
Volume	1000 ml
Apparatus	2 Paddle
Speed	50 rpm
Temp	37ºC
Time interval	(10,15,20,30,45)
Detection	HPLC (210 nm)

### Dissolution profile comparison

Moore and Flanner proposed a model independent mathematical approach to compare the dissolution profile using two factors, difference factor (f1) and similarity factor (f2)^22^. To evaluate the in vitro pharmaceutical equivalence, mean dissolution values were employed to calculate f1 and f2. The following equations were used to calculate f1 and f2 for the tested batches:

$$ {\text{f}}1 = \left\{ {\frac{{\sum\nolimits_{t = 1}^{n} {\left| {Rt - Tt} \right|} }}{{\sum\nolimits_{t = 1}^{n} {Rt} }}} \right\} \times 100 $$

$$ {\text{f2}} = 50\log \left\{ {\left[ {1 + \left( {\frac{1}{{\text{n}}}} \right)\sum\nolimits_{t = 1}^{n} {({\text{Rt}} - {\text{Tt}})^{2} } } \right]^{ - 0.5} 100} \right\} $$

Where n is the number of time points, Rt is the dissolution value of the reference product at time t, and Tt is the dissolution value of the test product at time t^22^.

## Conclusion

A dissolution test for six generic vildagliptin tablets was evaluated and presented in this study. The current study presents an in vitro dissolution study for vildagliptin for the first time in Saudi Arabia. The dissolution profile of tested vildagliptin samples showed significant similarity to the reference drug and meets the FDA guidelines. Such study could be considered to present a quality control tool for generic drugs to enhance post-production quality control.

## Data Availability

The datasets used and/or analysed during the current study available from the corresponding author on reasonable request.
